# Age- and sex-related differences of periodontal bone resorption, cognitive function, and immune state in APP/PS1 murine model of Alzheimer’s disease

**DOI:** 10.1186/s12974-023-02790-1

**Published:** 2023-06-27

**Authors:** Huiwen Chen, Yue Liao, Xu Zhang, Hui Shen, Dihua Shang, Zhiyan He, Wei Zhou, Zhongchen Song

**Affiliations:** 1 Department of Periodontology, Shanghai Ninth People’s Hospital, Shanghai Jiao Tong University School of Medicine; College of Stomatology, Shanghai Jiao Tong University; National Center for Stomatology; National Clinical Research Center for Oral Diseases, Shanghai Key Laboratory of Stomatology; Shanghai Research Institute of Stomatology, Shanghai, 200011 China; 2 Laboratory of Oral Microbiota and Systemic Diseases, Shanghai Ninth People’s Hospital, Shanghai Jiao Tong University School of Medicine; College of Stomatology, Shanghai Jiao Tong University; National Center for Stomatology; National Clinical Research Center for Oral Diseases, Shanghai Key Laboratory of Stomatology; Shanghai Research Institute of Stomatology, Shanghai, 200011 China

**Keywords:** Alzheimer’s disease, APP/PS1, Alveolar bone, CD4^+^T cells, Immune imbalance

## Abstract

**Background:**

The existence of an interconnected mechanism between cognitive disorders and periodontitis has been confirmed by mounting evidence. However, the role of age or sex differences in this mechanism has been less studied. This study aims to investigate sex and age differences in the characterization of periodontal bone tissue, immune state and cognitive function in amyloid precursor protein/presenilin 1(APP/PS1) murine model of Alzheimer’s disease (AD).

**Methods:**

Three- and twelve-month-old male and female APP/PS1 transgenic mice and wild-type (WT) littermates were used in this study. The Morris water maze (MWM) was used to assess cognitive function. The bone microarchitecture of the posterior maxillary alveolar bone was evaluated by microcomputed tomography (micro-CT). Pathological changes in periodontal bone tissue were observed by histological chemistry. The proportions of helper T cells1 (Th1), Th2, Th17 and regulatory T cells (Tregs) in the peripheral blood mononuclear cells (PBMCs) and brain samples were assessed by flow cytometry.

**Results:**

The learning ability and spatial memory of 12-month-old APP/PS1 mice was severely damaged. The changes in cognitive function were only correlated with age and genotype, regardless of sex. The 12-month-old APP/PS1 female mice exhibited markedly periodontal bone degeneration, evidenced by the decreased bone volume/total volume (BV/TV), trabecular thickness (Tb.Th), and bone mineral density (BMD), and the increased trabecular separation (Tb.Sp). The altered periodontal bone microarchitecture was associated with genotype, age and females. The flow cytometry data showed the increased Th1 and Th17 cells and the decreased Th2 cells in the brain and PBMC samples of 12-month-old APP/PS1 mice, compared to age- and sex-matched WT mice. However, there was no statistical correlation between age or sex and this immune state.

**Conclusions:**

Our data emphasize that age and sex are important variables to consider in evaluating periodontal bone tissue of APP/PS1 mice, and the cognitive impairment is more related to age. In addition, immune dysregulation (Th1, Th2, and Th17 cells) was found in the brain tissue and PBMCs of APP/PS1 mice, but this alteration of immune state was not statistically correlated with sex or age.

**Supplementary Information:**

The online version contains supplementary material available at 10.1186/s12974-023-02790-1.

## Introduction

Periodontitis is a chronic inflammatory disease characterized by the destruction of the periodontium [1]. Recent evidence has indicated that there is an unambiguous relationship between periodontitis and systemic disease. The potential impact of many systemic disorders (including diabetes mellitus, atherosclerosis, and respiratory disease) on the periodontium is well-documented [2].

Alzheimer’s disease (AD) is the most common form of dementia. It is a multifactorial progressive degenerative disorder that mainly affects aged people. Numerous epidemiological investigations and clinical observations have shown that periodontal infection might damage the central nervous system [3]. In our previous studies, *P. gingivalis*-LPS, one of the main virulence factors of *P. gingivalis*, led to learning and memory impairment in mice with intraperitoneal injection and rats with injection in the gingival sulcus [4]. However, the effect of AD on the periodontium has not yet been studied.

Imbalance in immune homeostasis is a common causative factor in periodontitis and AD [6]. In the activated immune system, CD4^+^T cells can differentiate into multiple subtypes, including helper T cells1 (Th1), Th2, Th17 and regulatory T cells (Tregs) [8]. They all have important implications for the development of diseases, such as periodontal disease and AD [9]. The balance between these subtypes is one of the keys to maintaining a normal immune response. Th1 and Th17 cells mediate inflammatory responses and autoimmune diseases; in contrast, Th2 and Treg cells have anti-inflammatory responses and maintain autoimmune tolerance [11]. Machhi et al. [12] reported that Aβ-reactive Th1 and Th17 effector T cells (Teffs), adoptively transferred into APP/PS1 mice demonstrated accelerated behavioral and pathological disease, thus supporting a role as disease perpetrators. However, there is no evidence whether these T cell-driven pro-inflammatory and peripheral immune activations would lead to periodontal tissue destruction.

Multiple studies have demonstrated sex differences in the prevalence, risk, and severity of AD patients [13]. Both epidemiological and clinical studies have suggested that women are more likely to develop AD than men, as have neuroimaging and postmortem human studies, which showed a stronger pathology in women than in men [14]. Previous studies on amyloid precursor protein/presenilin 1(APP/PS1) mice have mostly focused on a single sex. Considering that individual sex also has significant effects on bone homeostasis and the immune system, sex-related oral health and immune status differences remain an underappreciated and often overlooked issue. Therefore, there is a need to further elucidate the sexual dimorphism of periodontal characteristics and immune status in transgenic mouse models of AD.

In the present study, we report data from comparative studies using the Morris water maze (MWM) to assess spatial memory function in APP/PS1 mice of different sexes at 3 and 12 months of age. Histological assessment and micro-CT of the maxilla were performed upon completion of behavioral testing. The changes in the proportion of CD4^+^ T subsets in the blood and brain were assessed by flow cytometry. This study aims to investigate sex and age differences in the characterization of periodontal bone tissue, immune state and cognitive function in APP/PS1 murine model of AD. In addition, this study may reveal the damage caused by AD to the periodontium and its associated mechanisms of immune imbalance.

## Materials and methods

### Animals

Specific pathogen-free (SPF), male and female mice were used in the study. We used 3- and 12-month-old APP/PS1-mutant transgenic mice and age-matched nontransgenic wild-type (WT) littermates in this work. All animals were obtained from the Nanjing University Institute of Biomedical Sciences (Nanjing, China). The transgenes were confirmed by PCR genotyping of mouse tail tissue. All animal experimental protocols were approved by the ethics committee of Animal Care and Experimental Committee of Ninth People’s Hospital affiliated to Shanghai Jiao Tong University of Medicine (SH9H-2019-A499-1) and were carried out under the Guidelines from the EU Directive 2010/63/EU.

### Study design

The mice were divided into two age subgroups: 3 months of age and 12 months of age. The APP/PS1 mice in each subgroup were divided into male and female groups. Age- and sex-matched littermate WT mice were used as controls (*n* = 5, per group). The experimental animals were housed in a temperature- and humidity-controlled room under a 12-h light/dark cycle and were given free access to food and water. Prior to commencement of the experiments, the mice were allowed to acclimatize to their environment for 1 week. After the MWM tests were performed, all mice were sacrificed with sodium pentobarbital (30 mg/kg, intraperitoneal injection) according to the Panel on Euthanasia of the American Veterinary Medical Association. Maxillary bone was dissected, and then analyzed by microcomputed tomography (micro-CT) and histological analysis. Brain tissues and PBMCs were collected for flow cytometry.

### Morris water maze

The MWM equipment (Datum Mobile, Minhang, Shanghai, China) consisted of a black circular pool (with a diameter of 120 cm and a depth of 50 cm), a black platform (9 cm in diameter) and a video analysis system. The pool was filled with water until the platform in the pool was submerged 1 cm below the surface. The water temperature was held at 22 ± 1 ℃. The MWM test was performed in a dark room. The mice were released randomly with their heads facing toward the pool wall. They were given 90 s to learn to use the visual tips around the pool to find the hidden platforms. If the mouse failed to find the platform within 90 s, it was guided to the platform and allowed to stay there for 30 s [12].

Each mouse was trained for 4 times per day, and each training interval was 30 s. During the training, the time to find the platform was recorded as the latency. The probe trial was carried out on the sixth day to estimate retention performance. The platform was removed and the mice performed two trials in which they were placed in the two quadrants away from the platform and allowed to swim for 60 s [12]. The number of platform crossings and the time spent in the target quadrant by the mice were monitored and analyzed by the recording system in each trial.

### Microcomputed tomography

The excised maxillae were placed with gauze in the sample holder and scanned with a benchtop micro-CT imager (SkyScan 1076; Bruker-MicroCT, USA) at 9 μm resolution, 49 kV, and 179 μA. Bundled vendor software (CTAN software) was used for three-dimensional reconstruction and data processing. The measured structural parameters including bone volume/tissue volume (BV/TV; %), trabecular number (Tb.N; mm^−1^), trabecular thickness (Tb.Th; mm), trabecular separation (Tb.Sp; mm) and bone mineral density (BMD; g/cc) were analyzed to evaluate bone quality and resorption. The region of interest (ROI) was selected in the maxillary first molar area.

### Histological analyses

Following micro-CT analyses, the maxillae samples were exposed to decalcifying solution (10% EDTA), which was replaced every 3 days for 1 month, dehydrated, and then embedded in paraffin. Maxillary sections were made from the mesio-distal direction. Serial sections of 4 μm in thickness were cut. A hematoxylin–eosin (H&E) staining assay was used in accordance with the manufacturer’s instructions (Beyotime, Shanghai, China).

### Flow cytometry

Brain tissues were mechanically ground and lymphocytes were extracted with Percoll (GE Healthcare Life) [15]. Whole blood was collected in a 5-ml EP tube containing EDTA [16]. PBMCs were isolated from anticoagulant blood samples using Ficoll (Biosera). All cells were resuspended in RPMI-1640 medium (Gibco) containing 10% heat-inactivated fetal bovine serum, 100 U/ml penicillin, and 200 mM l-glutamine at 37 ℃ in a 5% CO_2_ environment. To stain for the intracellular cytokines IFN-γ, IL-4, and IL-17A, cell suspensions were stimulated for 5 h in the presence of GolgiPlug (BD Pharmingen, Cat. No. 550583). The cells were fixed, permeabilized, and stained according to the manufacturer’s instructions at 4 °C in the dark, and the data were analyzed by NovoExpress V1.4.0.

### FACS antibodies

The cells were blocked with anti-CD16/32 (BD Pharmingen, Cat. No. 553141) to reduce nonspecific binding.

Antibodies for FACS included FITC-labeled anti-mouse CD4 antibody (BD Pharmingen, Cat. No. 553650), PE-labeled anti-mouse INF-γ antibody (BD Pharmingen, Cat. No. 554412), PE-Cy™7-labeled anti-mouse IL-4 antibody (BD Pharmingen, Cat. No. 560699), Alexa Fluor® 647-labeled anti-mouse IL-17A antibody (BD Pharmingen Cat. No. 560184), PE-labeled anti-mouse CD25 antibody (BD Pharmingen, Cat. No. 553075), and Alexa Fluor® 647-labeled anti-mouse Foxp3 antibody (BD Pharmingen, Cat. No. 560401).

The cells were stained with CD4, INF-γ, IL-4, and IL-17A antibodies to define Th1, Th2, and Th17 cells and CD4, CD25, and Foxp3 antibodies to define Treg cells.

### Statistical analysis

All experiments were repeated at least three times independently. The data are expressed as mean ± standard error of the mean (SEM). The statistical analysis involved repeated measures ANOVA, one-way ANOVA, and Student’s *t* test with GraphPad Prism software V9.4.0 (GraphPad Software, Inc., La Jolla, CA, USA). All hypothesis tests were conducted at a significance level of 0.05.

## Result

### Distinct spatial learning and memory changes in the MWM test

#### Spatial learning

**Fig. 1:**
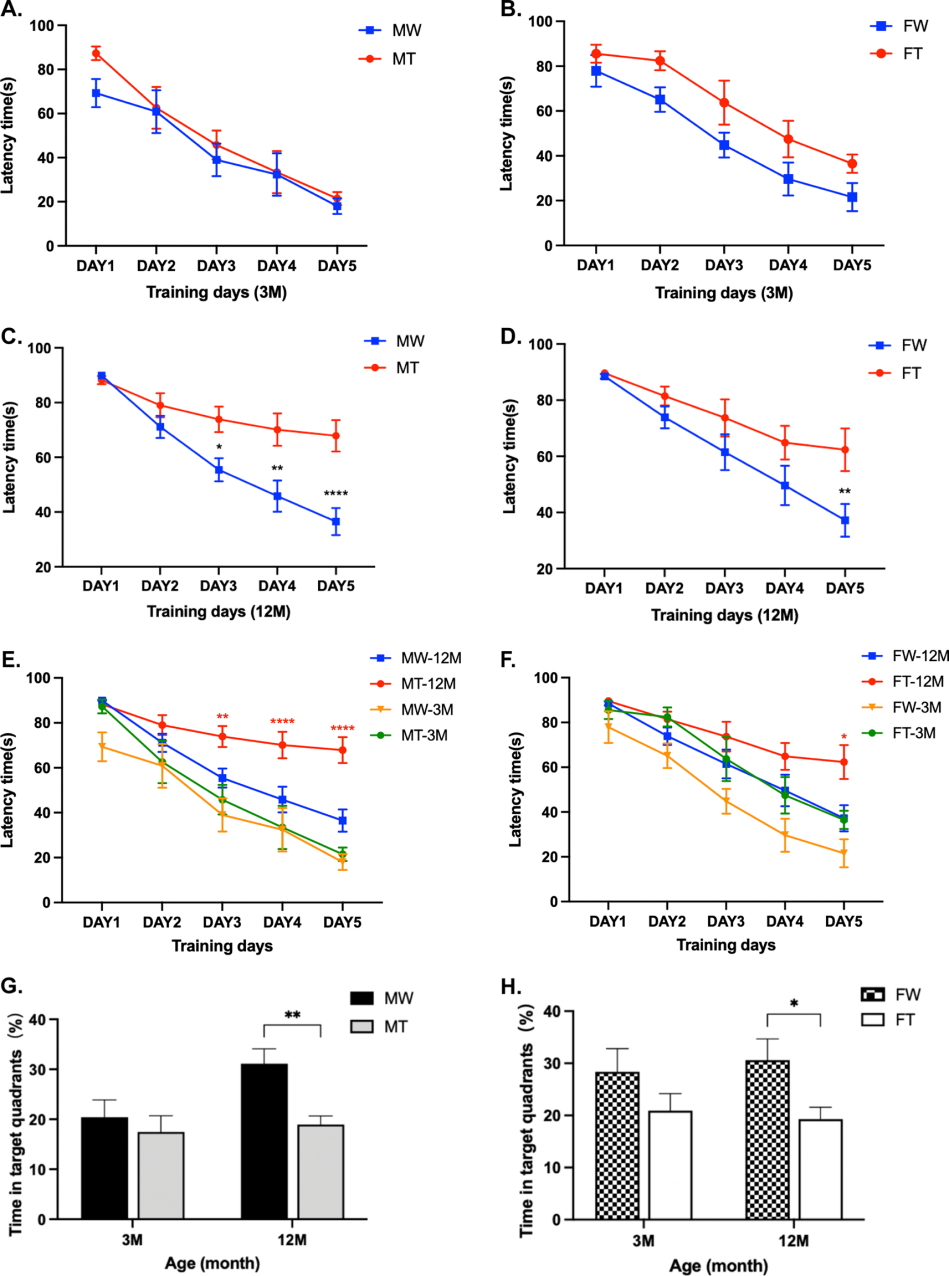

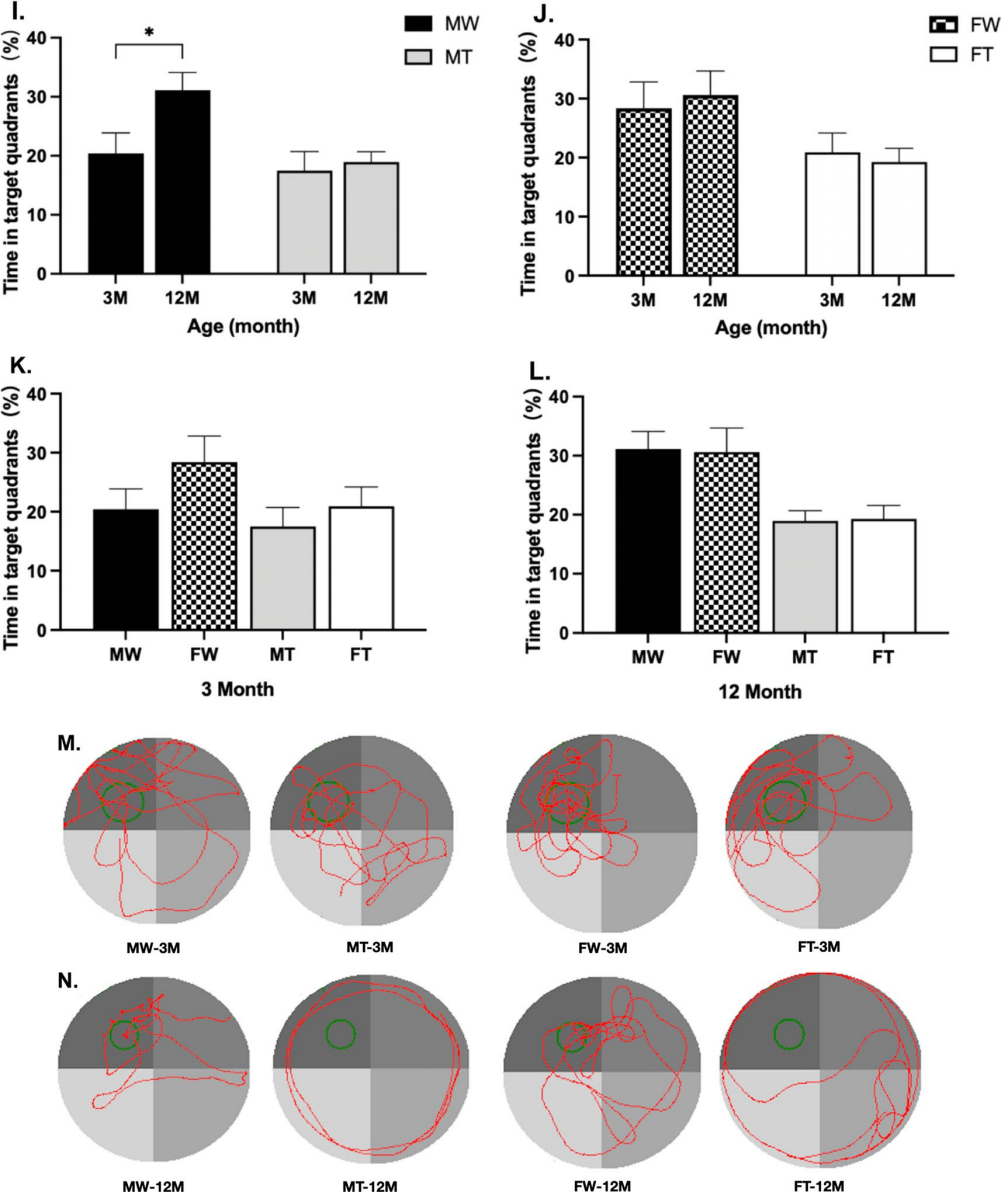
Learning and memory ability in 3- and 12-month-old male and female APP/PS1 mice and WT littermates. The Morris Water Maze test was conducted to assess the spatial learning and memory. **A–D** Difference in escape latency during the training phase between WT mice and APP/PS1 mice within the same age and sex (*P < 0.05, **P < 0.01, and ****P < 0.0001). **E,** **F** Difference in escape latency during the training phase between 12-month-old and 3-month-old mice within the same genotype and sex (*P < 0.05, **P < 0.01, and ****P < 0.0001). **G,** **H** Difference in the percentage of time in target quadrants between WT mice and APP/PS1 mice within the same age and sex. **I,** **J** Difference in the percentage of time in target quadrants between 12-month-old and 3-month-old mice within the same genotype and sex. **K,** **L** Difference in the percentage of time in target quadrants between males and females within the same genotype and age. (*P < 0.05, **P < 0.01, and ***P < 0.001). **M,** **N** Typical swimming trajectory.** (**MT: male APP/PS1 group; MW: male WT group; FT: female APP/PS1 group; FW: female WT group; 3 M: 3-month-old; 12 M: 12-month-old)

All mice were trained in the MWM task during a 5-day period. As shown in Fig. 1, the escape latency to find the platform showed a progressive decline. Repeated-measures ANOVA revealed no significant between-group effects in the latency between 3-month-old APP/PS1 mice and age-matched WT mice during all training days, regardless of sex (Fig. 1A, B) (*P* > 0.05). This result suggested that there was no impaired spatial learning in 3-month-old APP/PS1 mice. In comparison, 12-month-old APP/PS1 mice had significantly longer escape latencies than the matched WT mice. In male mice, the latency period of APP/PS1 mice began to be significantly longer than that of matched WT mice by the third day (*P* < 0.05) (Fig. 1C). Female APP/PS1 mice had a significantly longer latency on day 5 than matched WT mice (*P* < 0.01) (Fig. 1D).

There was no significant difference in escape latency between male and female mice of the same age and genotype, suggesting little effect of sex on spatial learning. In a comparison of the same genotype and sex, 12-month-old APP/PS1 mice had a longer escape latency than 3-month-old APP/PS1 mice regardless of sex. However, age had no effect on escape latency in WT mice (Fig. 1E, F). The above results illustrated that the learning ability of 12-month-old APP/PS1 mice was severely damaged.

#### Spatial memory

The spatial memory of all mice was evaluated by the probe trial performed 24 h after the last training session. The mean percentage searching time in the target quadrant and the mean percentage distance in the target quadrant represent spatial memory performance.

The 12-month-old APP/PS1 mice spent less time in the target quadrant than the age- and sex-matched WT mice (Fig. 1G, H). Furthermore, 12-month-old APP/PS1 mice traveled a significantly shorter distance in the target quadrant than those in the matched WT group (*P* < 0.01) (Additional file 1: Fig. S1A, S1B). However, these aforementioned differences were not observed in male and female mice at 3 months of age.

In the same-sex comparison at different ages, the 12-month-old WT male mice stayed in the target quadrant much longer than 3-month-old WT male mice (*P* < 0.05). Nevertheless, no such significant differences were observed in female mice and APP/PS1 male mice (Fig. 1I, J). In comparisons of the mean percentage distance in the target quadrant, 12-month-old mice were higher than 3-month-old mice in both sexes, with the elevation was much greater in WT mice than in APP/PS1 mice (Additional file 1: Fig. S1C, S1D).

In the same-age comparison across sexes, the searching time and the distance in the target quadrant in the same genotype of mice were independent of sex (Fig. 1K, L, Additional file 1: Fig. S1E, S1F).

The above results illustrated that spatial memory in 12-month-old APP/PS1 mice was severely impaired. The alteration of cognitive function is only correlated with age and genotype, not with gender. Figure 1M, N presents the swim tracks of representative animals.

### Decreased alveolar bone detected by micro-CT

**Fig. 2:**
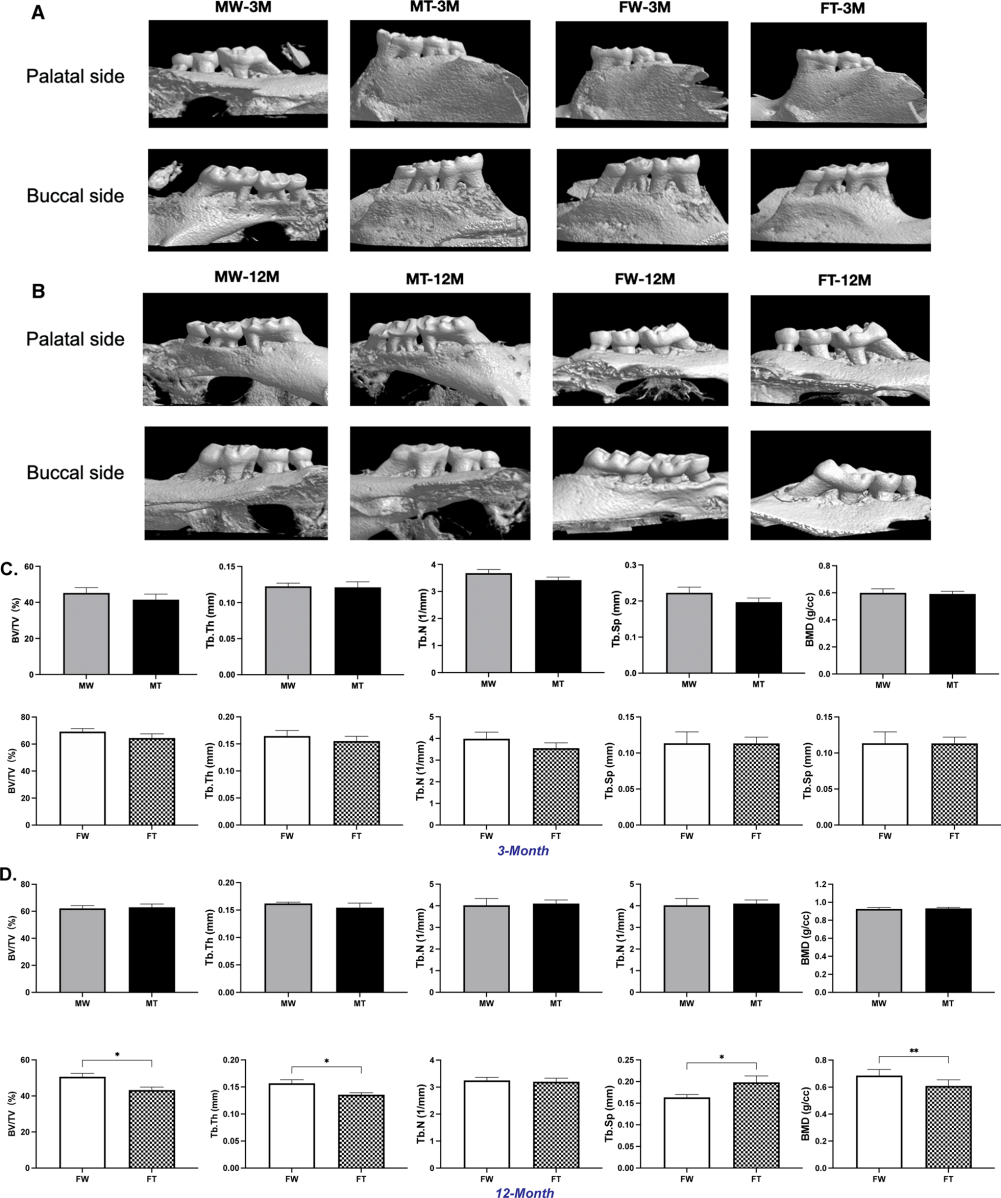

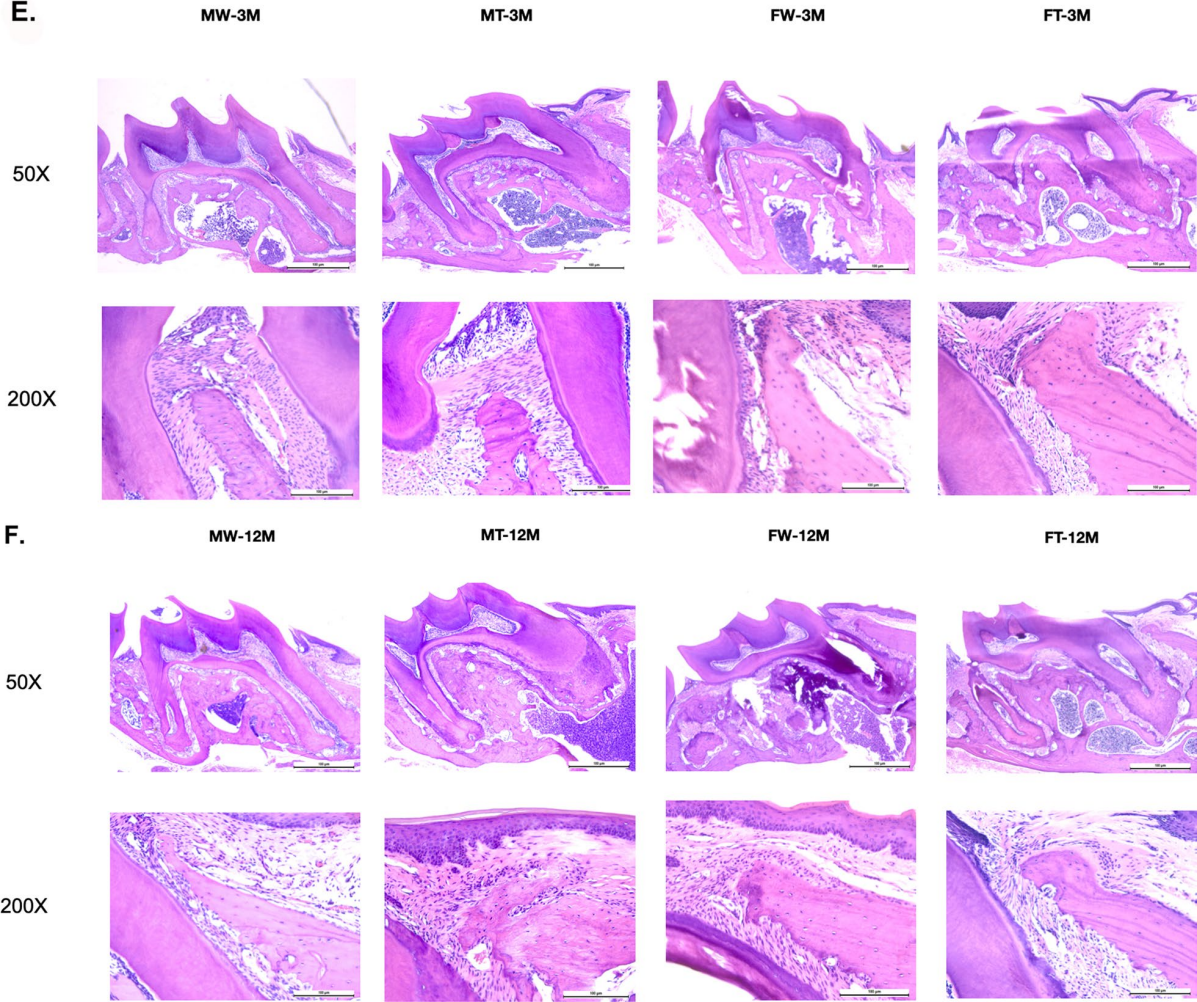
Periodontal tissue and alveolar bone in 3- and 12-month-old male and female APP/PS1 mice and WT littermates. **A** Micro-CT scanning of the molar region in 3-month-old mice. **B** Micro-CT scanning of the molar region in 12-month-old mice. **C** Alveolar bone parameter analysis of 3-month-old mice. **D** Alveolar bone parameter analysis of 12-month-old mice. **E** Maxillary alveolar bone of 3-month-old mice on H&E staining at a magnification of × 50 and × 200. **F** Maxillary alveolar bone of 12-month-old mice on H&E staining at a magnification of × 50 and × 200. **(**MT: male APP/PS1 group; MW: male WT group; FT: female APP/PS1 group; FW: female WT group; 3 M: 3-month-old; 12 M: 12-month-old)

Noticeable resorption of posterior maxillary alveolar bone was found in 12-month-old APP/PS1 female mice by micro-CT images but not in males. The bone loss even progressed to involve furcations (Fig. 2A, B). Compared with the parameters in the maxillae of the control group, the Tb.Sp (from 0.1633 ± 0.006686 to 0.1982 ± 0.01480 mm) was significantly increased in the APP/PS1 female group, and the BV/TV (from 41.20 ± 1.875 to 36.59 ± 1.638%), BMD (from 0.6861 ± 0.01562 to 0.6089 ± 0.01597 g/cc), and Tb.Th (from 0.1569 ± 0.006402 to 0.1356 ± 0.003860 mm) were markedly decreased (*P* < 0.05). Nevertheless, in 3-month-old mice (both male and female) and in 12-month-old APP/PS1 male mice, the differences in alveolar bone volume and bone microarchitecture were not evident (Fig. 2C, D).

In the same-sex comparison at different ages, with the increase of age, it can be found that the bone microstructure of 12-month-old male mice was superior to that of 3-month-old male mice. However, in females, the opposite is true. The BV/TV and Tb.N of 12-month-old female mice were inferior to those of 3-month-old female mice (Additional file 2: Fig. S2A).

In the same-age comparison, it could be found that the bone microstructure of 3-month-old females was superior to that of 3-month-old males, which was more evident in the WT mice. However, the situation was completely reversed in the 12-month-old mice, where the bone microstructure of 12-month-old females was much inferior to that of 12-month-old males, and this was more pronounced in APP/PS1 mice (Additional file 2: Fig. S2B). These results suggest that the altered periodontal bone microarchitecture is associated with genotype, age and females.

### Changes in periodontal tissue detected by histological analyses

To determine the pathological changes in APP/PS1 transgenic mice, we observed the posterior maxillary alveolar bone using histological chemistry. Hematoxylin and eosin (H&E) staining of periodontal tissue in the maxillary first molar is shown in Fig. 2E, F. In the 3-month-old mice, there was no significant difference in periodontal tissue among the four groups (Fig. 2E). In the 12-month-old mice, compared with those of sex-matched WT mice, apical migration of the junctional epithelium, dished absorption of molar roots, reduced bone height, irregular resorption lacunae, narrowed pulp cavity and thickened dentin were found in the APP/PS1 mice (Fig. 2F). The results indicated that 12-month-old APP/PS1 mice exhibited periodontal destruction and bone degeneration.

### Proportion of CD4^+^ T cells detected by flow cytometry

**Fig. 3:**
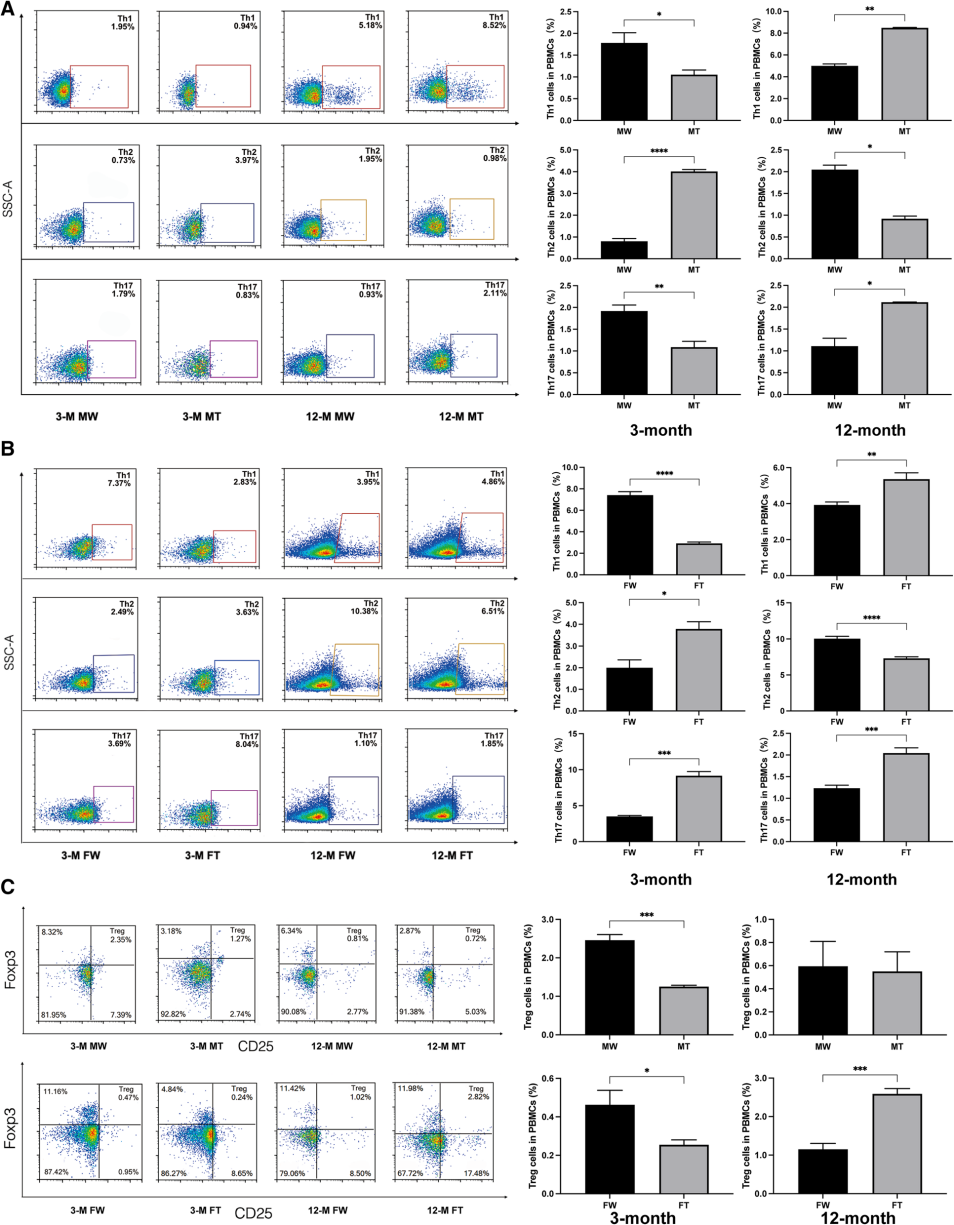
CD4^+^ T cells in PBMCs by flow cytometry in 3- and 12-month-old male and female APP/PS1 mice and WT littermates. The proportion of Th1, Th2, Th17, and Treg cells was determined. **A** Proportion of Th1, Th2, and Th17 in male mice. **B** Proportion of Th1, Th2, and Th17 in female mice. **C** Proportion of Tregs. (Student’s *t* test. *P < 0.05, **P < 0.01, ***P<0.001, and ****P < 0.0001 compared to the WT group) **(**MT: male APP/PS1 group; MW: male WT group; FT: female APP/PS1 group; FW: female WT group; 3 M: 3-month-old; 12 M: 12-month-old)

**Fig. 4:**
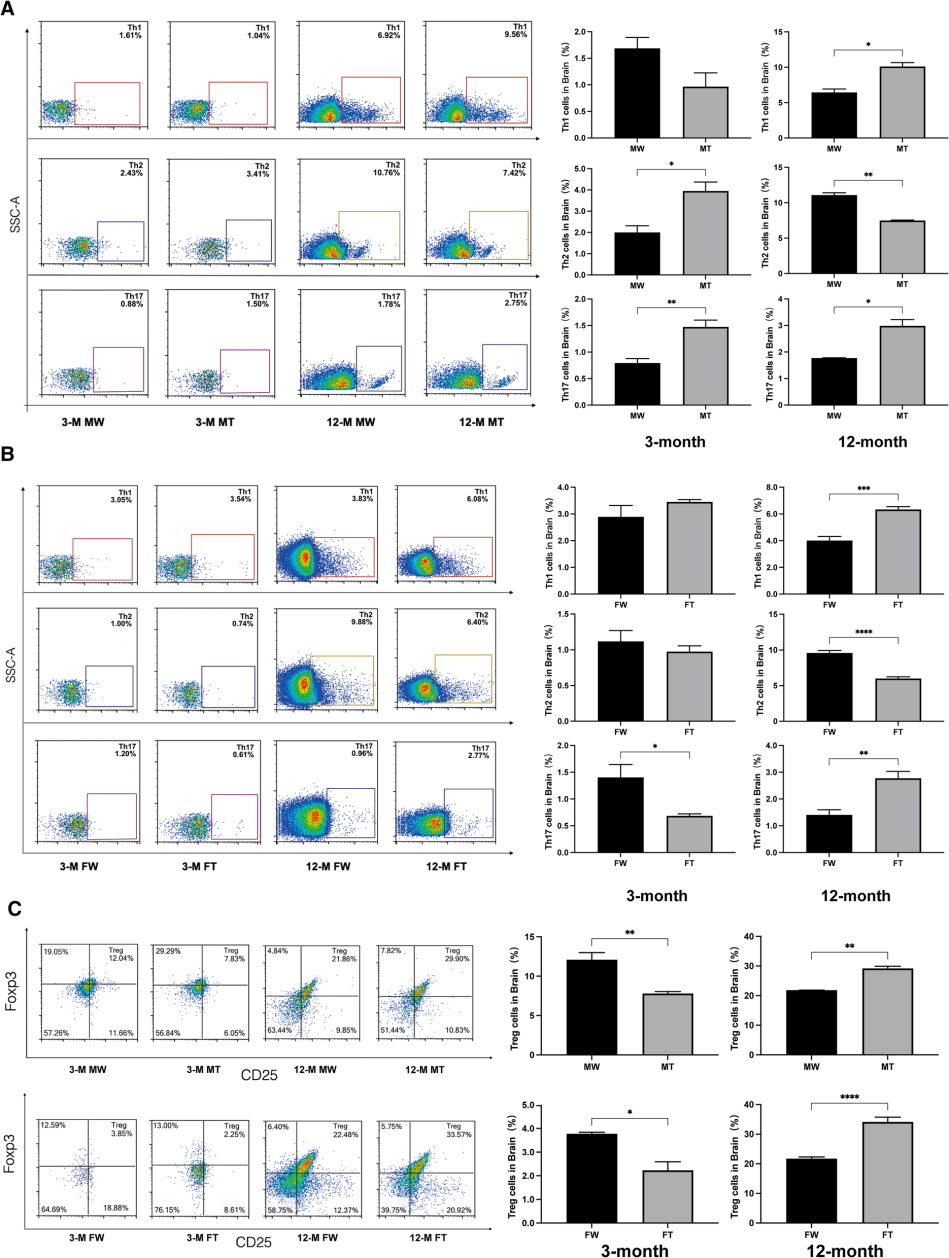
CD4^+^ T cells in brain by flow cytometry in 3- and 12-month-old male and female APP/PS1 mice and WT littermates. The proportion of Th1, Th2, Th17, and Treg cells was determined. **A** Proportion of Th1, Th2, and Th17 in male mice. **B** Proportion of Th1, Th2, and Th17 in female mice. **C** Proportion of Tregs. (Student’s *t* test. *P < 0.05, **P < 0.01, ***P<0.001, and ****P < 0.0001 compared to the WT group) **(**MT: male APP/PS1 group; MW: male WT group; FT: female APP/PS1 group; FW: female WT group; 3 M: 3-month-old; 12 M: 12-month-old)

In male mice, the expression of Th1 and Th17 cells in PBMCs was significantly upregulated in 12-month-old APP/PS1 mice, while the expression of Th2 decreased compared with that in age-matched WT mice (Fig. 3A, C). The expression of Th1, Th17 and Treg cells in the brain was significantly increased in 12-month-old APP/PS1 mice, while the expression of Th2 cells was decreased, compared with that in age-matched WT mice (Fig. 4A, C).

In female mice, the expression of Th1, Th17 and Treg cells in PBMCs was significantly upregulated in 12-month-old APP/PS1 mice, while the expression of Th2 cells decreased, compared with that of age-matched WT mice (Fig. 3B, C). Similar results were observed in the brain (Fig. 4B, C).

In different age comparisons between the same sex, the expression of Th1, Th2, Th17 and Tregs was significantly higher in the brain tissue of 12-month-old male mice compared to 3-month-old males, and a similar trend could be observed in females. However, in PBMCs, there were significant sex differences in this age comparison. In the same-age comparison across sex, in both brain tissue and PBMCs, the expression of Th1 cell was greater in 3-month-old female mice than in males, while Treg cell expression was greater in males than in females. In 12-month-old APP/PS1 mice, the expression of Th1 in PBMCs was greater in males than in females, but the expression of Th2 and Treg were smaller in males than in females. In addition, the expression of Th1 and Th2 cells were greater in the brain tissue of 12-month-old APP/PS1 male mice than in females (Additional file 3: Fig. S3 and Additional file 4: Fig. S4). However, in short, the above comparative results are too complicated to conclude that the immune status of APP/PS1 mice presents a direct correlation with age or sex.

## Discussion

The major result of the current study is that the pathological process of AD may cause impairment in the periodontium in the middle-aged mice, especially in alveolar bone in females, suggesting that periodontal bone resorption in AD mice is sex- and age-related. Homeostatic imbalance between regulatory T cells (e.g., Treg) and effector T cells (Th1, Th2, and Th17) may be the underlying mechanism. However, this immune state is independent of sex and age.

The association between Alzheimer’s disease and periodontitis has recently received increasing attention [17]. Whether there is a causal relationship between these two pathologies remains unknown. Alzheimer’s disease, the main cause of dementia in the adult population, is considered that neuroinflammation plays a fundamental role in its onset and progression. The current evidence suggests that periodontitis can induce or exacerbate neuroinflammation in AD [18]. Periodontal pathogens and the immuno-inflammatory host response in periodontitis may affect the brain function [19]. Clinical study has shown that the severity of periodontitis in Alzheimer's disease is associated with cognitive decline [21]. However, whether the pathological course of AD can mediate the periodontal tissue damage and if there are age and sex differences in this damage have not been reported in previous studies.

The APP/PS1 transgenic mouse is an ideal model to examine the underlying AD pathophysiology and cognitive ethology [22]. The mice were produced by coinjection of APPswe and PS1∆E9 vector, and confirmed by PCR genotyping using appropriate oligonucleotide primers. This model replicates the formation of amyloid-beta (Aβ) plaques in the mouse brain, with obvious learning and memory dysfunction at 8 months [23]. In the Morris water maze test in this study, it was also confirmed that both male and female APP/PS1 mice showed significantly impaired cognition at 12 months of age but not at 3 months of age. The alteration of cognitive function is only correlated with age and genotype, not with gender.

### Severe bone loss of the maxillae and histopathological changes in the periodontium in APP/PS1 transgenic mice

According to the published literature, an epidemiological correlation between AD pathogenesis and the loss of dental elements is supported by different cohort studies [24]. Patients suffering from AD are characterized by a greater number of lost teeth and general edentulism compared to the control groups [25]. In particular, Takeuchi et al. [26] showed a correlation between dental loss and dementia, with an inverse association between the number of residual dental elements and the risk of developing dementia, particularly AD. Apart from external factors such as trauma, alveolar bone resorption is the most important cause of tooth loss. In our study, compared with age-matched WT mice, bone loss was detected in the maxillae of 12-month-old APP/PS1 female mice by micro-CT. This result is consistent with the clinical findings. Patients with AD often have lower bone mass than healthy individuals [27]. In the histological examination, noticeable destruction of the periodontium in APP/PS1 mice was detected in the maxillary alveolar bone, including apical migration of the junctional epithelium and alveolar bone loss. Unlike the mouse model of periodontitis, the APP/PS1 mouse models in our study were never subjected to inoculation with *P*. *gingivalis* or molar ligation to induce periodontal bone loss and thus, the outcome cannot be explained by microbial infection. In summary, it is highly likely that alveolar bone resorption and impairment in the periodontium in APP/PS1 mice are related to the pathological process of AD.

### Imbalance of immune homeostasis in APP/PS1 transgenic mice

The homeostasis of the skeletal system depends on the dynamic balance between osteogenesis by osteoblasts and bone resorption by osteoclasts [28]. Shifting this balance in favor of osteoclast formation leads to pathological bone resorption. Studies of bone destruction associated with inflammatory bone disease have emphasized the importance of the interaction between the immune and skeletal systems, with local inflammatory mediators produced by lymphocytes playing a nonnegligible role in bone resorption. Recent studies have stressed neuroinflammation as a relevant mechanism in AD pathogenesis [29]. Various Th cell lineages, such as Th1, Th17, and Treg cells, have been described to be closely associated with AD [12]. Proinflammatory subpopulations, such as Th1 and Th17 cells, play an important contributing role in the physiopathology of AD [31]. In normal circumstances, the blood–brain barrier isolates the central nervous system from the systemic circulation to sustain an optimal microenvironment. Both in vivo and in vitro studies have shown that Th17 cells and their signature cytokine IL-17 could alter the permeability of the blood–brain barrier. This leads to barrier dysregulation and increased cellular circulation, promoting bidirectional crosstalk between brain tissue and the peripheral circulation [32]. In our study, the Th1 and Th17 cells were significantly increased, and the Th2 cells were significantly decreased in both brain tissue and PBMCs. This result suggested that this immune dysregulation (Th1, Th2, and Th17 cells) within the central nervous system of AD may cause an imbalance in the immune microenvironment of the systemic circulation, and immune inflammatory mediators of the brain could affect periodontal tissues through the peripheral blood circulation.

Periodontitis is a chronic bacterial infectious inflammatory disease in which plaque stimulates the host inflammatory response, leading to periodontal tissue damage and bone resorption. Although many studies in the last decades have focused on plaque, the major periodontal tissue destruction may be due to a series of immune inflammatory responses to bacteria [34]. Periodontal bone immunology is now attracting more attention as one of the causes of periodontal disease, and alveolar bone resorption can be caused directly or indirectly by cellular inflammatory infiltration [35]. In general, periodontal bone immunology implies that immune cells (e.g., T lymphocytes) can communicate molecularly with both types of effector bone cells [36]. It has been demonstrated that resorption of alveolar bone in periodontal lesions is associated with an elevated local RANKL/OPG ratio [34]. Among the T-cell subsets, Th1 lymphocytes directly produce IL-1β, IL-12 and IFN-γ, which exert osteoclast activity indirectly by inducing enhanced RANKL expression on osteoblasts and Th17 lymphocytes. Th17 lymphocytes can induce osteoclast differentiation and activation by producing RANKL. In addition, IL-17A produced by Th17 lymphocytes can recruit inflammatory cytokines such as TNF-α and IL-6 to enhance local inflammation and further promote the activity and expression of RANKL [37]. In contrast, Th2 and Treg cells, as anti-inflammatory subpopulations, can produce the anti-inflammatory factors IL-4 and IL-10 to inhibit osteoclastogenesis, reduce RANKL activity, maintain periodontal bone immune homeostasis, and control immune-mediated bone tissue damage [35]. In the present study, the enhanced ratio of Th1 and Th17 cells and diminished ratio of Th2 cells in the peripheral circulation may be one of the main causes of periodontal inflammatory bone loss.

### Sexual dimorphism in periodontal bone homeostasis and immunity in APP/PS1 transgenic mice

It is increasingly important to acknowledge sex differences in immune responses and bone homeostasis. Since 2015, the field of sex-based biology has launched several new policies to promote more consideration, reporting and analysis of sex in biomedical science to improve the rigor of research [39].

Sex is one of the major risk factors for AD, with a higher incidence of the disease in females [41]. Osteoporosis is a common complication in women with AD, with patients exhibiting reduced mineralization and risk of fragility fractures [42]. Estrogen deficiency, in light of menopause, has distinctly been recognized as a major risk factor for osteoporosis in women [42]. The effects of estrogen on the skeleton have been the focus of intense investigation. Studies are strongly suggestive that sex steroids impactfully modulate the host immune response and bone homeostasis, which raises the potential for alterations in the course of destructive periodontal disease [43]. Evidence demonstrates that estrogen deficiency is associated with not only the rapid alveolar bone loss associated with early menopause but also with the later slow phase of alveolar bone loss attributed to aging [44]. Adverse changes in bone density or quality are linked to greater susceptibility to destructive periodontal disease. A cross-sectional evaluation suggests that low systemic bone mineral density is associated with worse periodontal disease measures [45]. Another study also confirmed an association between osteoporosis and reduced alveolar crest height in postmenopausal women [46]. In the present study, 12-month-old female APP/PS1 mice exhibited more pronounced alveolar bone destruction, specifically a decrease in bone volume and bone trabecular thickness. This result is similar to the bone changes in postmenopausal women. In contrast, bone density in aged male mice was more conservative, consistent with clinical epidemiological findings [47].

Estrogen regulates elements related to immune function and bone metabolism, which is reflected in the activation of osteoclasts by T cells. The influence of estrogen on the T-cell response is biphasic. Estrogen has been shown to affect cytokine production of both the type 1 and type 2 helper T-cell subsets [48]. In animal and human pregnancy studies, there is a shift from the Th1 to the Th2 immune response presumably partly driven by sex hormones [49]. The provocation of bone resorption in response to estrogen deficiency appears mainly to be the result of a cytokine-driven increase in osteoclast formation. Estrogen induces the formation of osteoclastogenic factors by activated T cells [43]. However, in the present findings, it is difficult to conclude evidence that the immune imbalance within the brain tissue and PBMCs of APP/PS1 mice is correlated with sex.

## Conclusions

Age and sex are important variables to consider in evaluating periodontal bone tissue, and the cognitive impairment of APP/PS1 mice is more related to age. Cognitively impaired 12-month-old APP/PS1 female mice had more impaired periodontal tissues (especially alveolar bone) compared to age- and sex-matched WT mice. In addition, immune dysregulation (Th1, Th2 and Th17 cells) was found in the brain tissue and PBMCs of APP/PS1 mice, but this alteration of immune state was not statistically related to sex or age. The imbalance in immune homeostasis may be the mechanism of the impact of AD on the destruction of periodontal bone, while this hypothesis still needs to be further confirmed in individual mice. More experiments are needed in the future to prove that there is a correlation mechanism between AD immune inflammation and periodontal conditions.

## Supplementary Information

**Additional file 1**: **Figure S1**. Percentage of distance in the target quadrant. A, B Difference between WT mice and APP/PS1 mice within the same age and sex. C, D Difference between 12-month-old and 3-month-old mice within the same genotype and sex. E, F Difference between males and females within the same genotype and age.

**Additional file 2**: **Figure S2**. Assessment of bone microstructure in molar region by micro CT. A. The difference between 12-month-old and 3-month-old mice within the same genotype and sex. B. The difference between males and females within the same genotype and age.

**Additional file 3**: **Figure S3**. CD4^+^ T cells in PBMCs by flow cytometry. A. The difference between 12-month-old and 3-month-old mice within the same genotype and sex. B. The difference between males and females within the same genotype and age.

**Additional file 4**: **Figure S4**. CD4^+^ T cells in brain tissues by flow cytometry. A. The difference between 12-month-old and 3-month-old mice within the same genotype and sex. B. The difference between males and females within the same genotype and age.

## Data Availability

The data sets and materials supporting the conclusions of this article are included within the article.

## Abbreviations

AD: Alzheimer's disease
APP/PS1: Amyloid precursor protein/presenilin 1
WT: Wild type
MWM: Morris water maze
Micro-CT: Micro-computed tomography
Th cells: Helper T cells
Tregs: Regulatory T cells
PBMCs: Peripheral blood mononuclear cells
SPF: Specific pathogen-free
PCR: Polymerase chain reaction
BV/TV: Bone volume/tissue volume
Tb.N: Trabecular number
Tb.Th: Trabecular thickness
Tb.Sp: Trabecular separation
BMD: Bone mineral density
ROI: Region of interest
EDTA: Ethylene diamine tetraacetic acid
H&E: Hematoxylin–eosin
IFN-γ: Interferon-γ
IL: Interleukin
FACS: Fluorescence activated cell sorting
SEM: Standard error of the mean
ANOVA: Analysis of variance
