# Supplementary figures and images for: Age- and sex-related differences of periodontal bone resorption, cognitive function, and immune state in APP/PS1 murine model of Alzheimer’s disease
Source: J Neuroinflammation. 2023 Jun 27;20:153. doi: 10.1186/s12974-023-02790-1 (PMC10294321; doi:10.1186/s12974-023-02790-1)

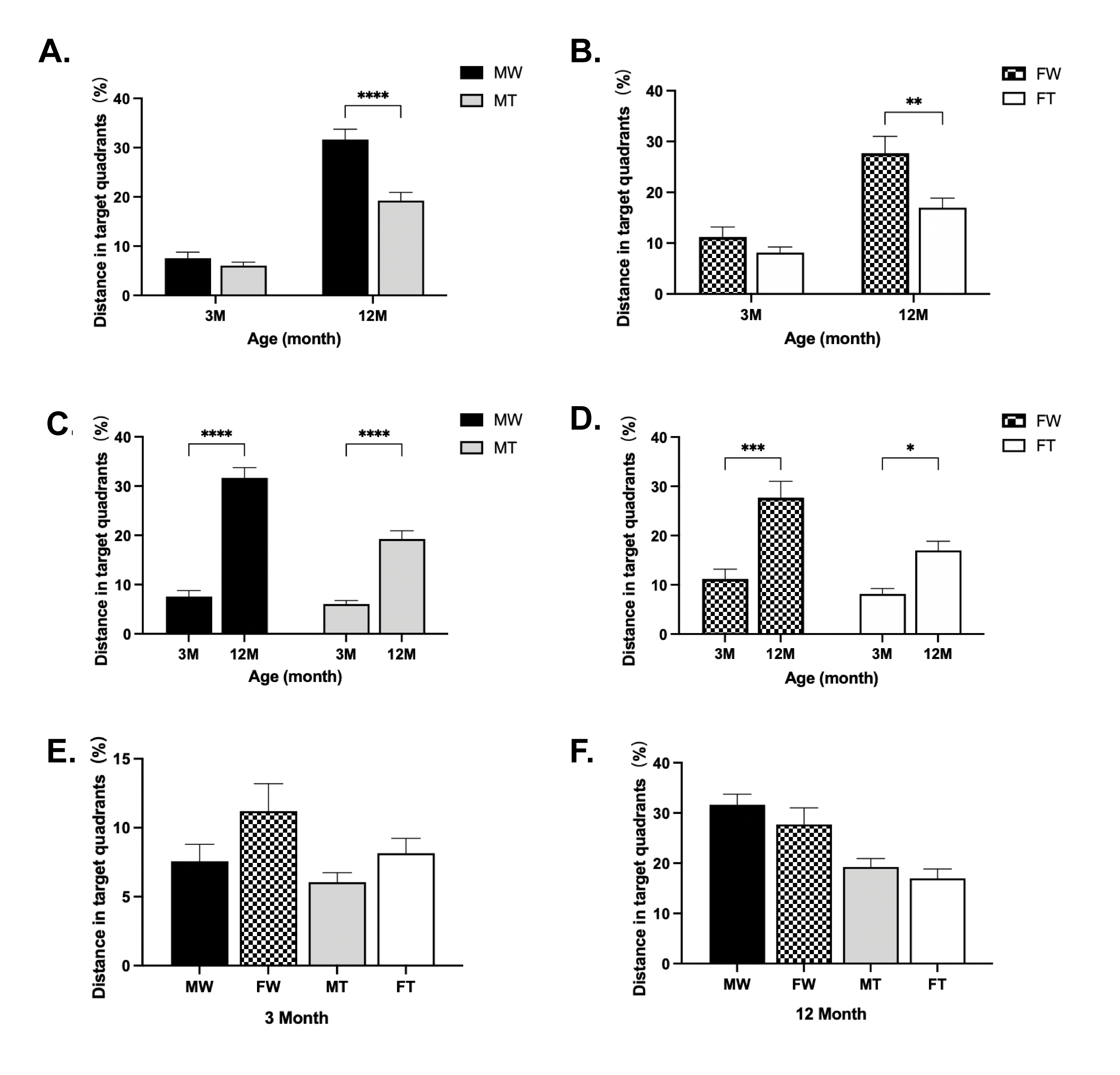

Supplement: Supplementary file 1 — Additional file 1: Figure S1. Percentage of distance in the target quadrant. A, B Difference between WT mice and APP/PS1 mice within the same age and sex. C, D Difference between 12-month-old and 3-month-old mice within the same genotype and sex. E, F Difference between males and females within the same genotype and age. [file 12974_2023_2790_MOESM1_ESM.tif]
